# Association of Family Nutrition and Physical Activity with Preschooler’s Working Memory: A Cross-Sectional Study among Mexican Children

**DOI:** 10.3390/children8060506

**Published:** 2021-06-15

**Authors:** Liliana Aguayo, Maria Pineros-Leano, Rifat B. Alam, Rosalba Aguirre-Pereyra, Andiara Schwingel, Solveig A. Cunningham

**Affiliations:** 1 Hubert Department of Global Health, Rollins School of Public Health, Emory University, Atlanta, GA 30322, USA; sargese@emory.edu; 2 School of Social Work, Boston College, Chestnut Hill, MA 02467, USA; pinerosl@bc.edu; 3 Department of Kinesiology and Community Health, University of Illinois at Urbana-Champaign, Champaign, IL 61820, USA; rifatalam.hf@gmail.com (R.B.A.); andiara@illinois.edu (A.S.); 4 Secretary of Education of the Government of the State of San Luis Potosi (SEGE), San Luis Potosi 78369, SLP, Mexico; jorgeferretisross@gmail.com

**Keywords:** family nutrition, physical activity, health behaviors, Mexican children, working memory

## Abstract

Healthy eating and active lifestyles are associated with children’s healthy weight and cognitive development. This study examines whether family behaviors relevant for nutrition and activity levels are associated with children’s working memory, independent of their weight status. A convenience sample of child–caregiver dyads (*n* = 85 dyads) were recruited from a public preschool serving a low-income community in central Mexico. Caregivers reported the frequency of ten family behaviors using the Family Nutrition and Physical Activity screening tool. Children completed a test of their ability to recall four words after a 60-s distraction task, an assessment of working memory. Multiple linear regression models were used to test the association of children’s working memory with each family behavior, adjusting for children’s sex, age, mother’s age and education, and subjective social status and then also adjusting for children’s age- and sex-specific body mass index percentile (BMI-P) and covariates. Higher frequency of breakfast intake was significantly associated with working memory (β = 0.57, *p* = 0.013). This association was independent of children’s BMI-P. Other family behaviors (frequent family mealtimes, limiting screen time, and others) were not significantly associated with children’s working memory. Frequent breakfast intake could benefit young children’s working memory, regardless of their weight status. This association merits further investigation.

## 1. Introduction

In Mexico, about 4% of children under age 5 are underweight, while over 30% have overweight or obesity [1]. Both underweight and obesity in early childhood are serious conditions that increase the risks for the development of cardiovascular disease, type 2 diabetes mellitus, cancer, and other chronic diseases in adulthood [2,3]. Observational and experimental studies have shown that both underweight and obesity are associated with poor cognitive function in children and adolescents, affecting executive functioning, self-regulation, attention, gross motor skill development, and visual/spatial performance [4,5,6,7].

In contrast, breakfast intake, active play, physical activity, and sufficient sleep are associated with healthier weight gain trajectories during childhood, optimal brain development, the ability to concentrate, and proficient academic performance [5,8,9,10].

A systematic review that summarized 45 intervention studies among children aged 3–20 years suggested breakfast intake has positive effects on children’s working memory and attention [9]. Frequent intake of fruits and vegetables and limited intake of snack foods have also been associated with executive cognitive function among fourth-grade children [11,12]. Among children aged 5 years old and younger, studies have shown that physical activity is positively associated with brain development, executive function, self-regulation, and other dimensions of children’s cognitive health [5].

Working memory is a component of children’s neurodevelopment and an important predictor of future cognitive functions and school achievement [13,14]. Early measurement of working memory and neurodevelopmental and behavioral problems are important for timely and effective interventions [13,15,16]. Assessment of working memory often involves sequential increases in the number of items that can be recalled; these item lists are believed to be processed or stored in the individual’s short-term or working memory [14,17]. Early childhood ages are particularly considered a critical period for the development of working memory as children are expected to have as much as twofold increases in their ability to perform in delayed recall test (tests after timed intervals or distractions) from the ages of 5 to 10 years [18]. Yet, to our knowledge, the association of family lifestyle behaviors with cognitive functions, particularly working memory in early childhood ages, has not been examined. This research can inform how families and programs can support children’s cognitive development, and how children’s weight status should be considered in cognitive development.

The benefits of healthy eating and physical activity practices for children’s cognition, such as executive function, attention, motor development, concentration, and academic proficiency may be moderated by children’s weight status [4,8]. It is not yet clear whether this association is independent of children’s weight [4]. It is important to better understand the contribution to children’s cognitive neurodevelopment introduced by lifestyle behaviors, especially by health behaviors that develop in family contexts at early ages. Research on childhood growth and risks of obesity has recognized the importance of family environments and caregivers, particularly during early childhood [19,20,21]. The family environment provides resources and opportunities that can limit or facilitate healthy growth and development [19]. In a longitudinal study of children followed from birth to 7 years of age, researchers observed that a range of family behaviors (e.g., frequency of breakfast and family meal times, participation in sports, visits to the park, and availability of unhealthy foods) explained between 7 and 11.3% of children’s weight [21]. Studies that examined family environments, including studies that measured these using the Family Nutrition and Physical Activity (FNPA) screening tool, have found that family environments are associated with children’s weight status, severe obesity, and 1-year change in body mass index percentile (BMI-P) [22,23,24,25,26].

The goal of this study is to explore the association between family nutrition and activity environments, as measured by the FNPA screening tool, and children’s ability to perform on a 60-s delayed recall test, a proxy measurement of working memory competences. The FNPA was developed to characterize “obesogenic” family environments for diverse samples of young children in the United States [22,23,24,25,26,27]. Using a cross-sectional and observational design with a sample of 4- to 6-year-old children living in San Luis Potosi, a city in central Mexico, this study has two aims: (1) to examine the association of family nutrition and physical activity environment with children’s performance on a working memory test and (2) to examine whether children’s BMI-P attenuates the association of family nutrition and physical activity environmental factors with children’s performance on the working memory test.

## 2. Methods

This study used data from the Family-based International Evaluation of Salivary Telomere-lengths and Acculturation (FIESTA) Study. FIESTA is a cross-sectional study that recruited children and their primary caregivers to examine the influence of maternal acculturation on salivary telomere lengths and adiposity. As part of the study, children in Mexico were asked to complete an assessment of working memory. All caregiver participants provided informed consent for themselves and assent for their children. Data were collected in 2016 during the months of September through November. All the procedures were approved by the University of Illinois at Urbana-Champaign Institutional Review Board, the Preschool’s Principal in Mexico, and the Secretaría de Educación de Gobierno del Estado (Secretary of Education of the Government of the State of San Luis Potosi).

### 2.1. Recruitment and Eligibility

Participants were recruited using flyers that were distributed among all children attending a public preschool in a low-income urban area where the study was conducted. Primary caregivers were eligible to participate if they were born in Mexico, aged 18 years old or older, spoke Spanish, had no physical or medical conditions, did not have a cognitive impairment that would affect their participation, and were the primary caregiver of a 4- to 6-year-old child who lived in their home and attended the preschool where the study was conducted. Children were eligible to participate if they were 4- to 6-years-old, attended the preschool where the study took place, and had no diagnosed physical or cognitive impairment.

### 2.2. Data Collection Procedures and Measures

Primary caregivers completed a self-administered questionnaire that collected information on family lifestyle behaviors and demographic characteristics of themselves and their 4- to 6-year-old child.

Family lifestyle behaviors. Primary caregivers were asked to report the frequency of ten family lifestyle factors (e.g., frequency of breakfast intake, family mealtimes) using the Family Nutrition and Physical Activity (FNPA) screening tool. This questionnaire includes 20 items designed to examine the frequency of ten factors associated with childhood obesity (two items examine each factor) and is validated among Hispanic/Latino populations in the US [27]. Table 1 introduces the specific items examined within each family lifestyle construct. Items are scored using a four-point Likert scale that ranges from never/almost never (score of 1) to very often/always (score of 4). Higher scores imply a more favorable family environment and lifestyle behaviors. Following instrument recommendations, we computed a score for each of the ten constructs from the sum of both items and an overall score from the sum of all items. The maximum possible overall total score was 80.

Children’s body mass index percentile (BMI-P). Child’s body weight was measured to the nearest 0.1 kg using the Inbody230™ (Biospace Ltd., Seoul, Korea) body composition analyzer. Children’s height was measured to the nearest 0.1 cm using a portable stadiometer (Seca 213, Hamburg, Germany). Age, sex, body weight, and height measurements were used to calculate child BMI-P. Center for Disease Control and Prevention (CDC) guidelines were used to determine child BMI-P and weight status categories because the reference population included children of Mexican descent and this approach facilitates future comparisons with Mexican American children. Children’s BMI-P was entered continuously in the analytical models.

Children’s working memory. An assessment of recall performance was used to examine children’s working memory. Cognitive function and working memory were not the primary focus of the FIESTA study. However, when designing the data collection, researchers consulted with a neurodevelopmental expert who recommended the delayed recall examination given the age of the sample, the limited availability of instruments appropriate for samples of young Mexican children, the low costs to researchers, and the low burden to participants. To select the working memory test, researchers reviewed standardized assessments of memory for children in the 3–6-year age range [14]. Researchers decided to introduce a working memory assessment, which consisted of asking children to repeat four words immediately and recall them after a timed distracting task [14]. Specifically, children were asked to recall the words *leche* (milk), *sol* (sun), *libro* (book), and *mostaza* (mustard). These words were selected given their high frequency of use, which would guarantee all children had previously heard these words, independent of their families’ level of education and socioeconomic status. The 60-s distracting task was filled with a word retrieval task where children had to elicit words in response to “things people eat”. A timer with an alarm was set to count the 60-s time interval. Working memory scores were calculated as the number of correctly recalled words. This assessment is appropriate for children in this age group and has been previously used in experimental studies with children as young as 4 years old to examine working memory and the role of attention in the maintenance of information [28].

All tests were conducted in a private office in the preschool’s library where only the child and the bilingual interviewer (LA) were present. To the extent possible, distractions and noise were limited while tests were being conducted. All appointments were schedule at 9:00 AM and children were asked to fast prior to their appointment. Upon arrival, children and caregivers completed the consent process, and child and caregivers’ height and weight were collected. Participants were asked to fast for the bioelectrical impedance assessments. Since children and caregivers were asked to fast for the assessments, both were provided with breakfast. Meal options included yogurt, corn cereal, whole milk, and fruits including bananas, oranges, and grapes. The assessment of recall was conducted after they finished eating their breakfast. Although the time that lapsed between children’s breakfast meal and working memory tests were very similar for most children, neither the time nor the foods consumed were assessed as part of this observational study.

Covariates. Statistical tests adjusted for the association of child’s sex and age and the primary caregiver’ age, years of completed education, and subjective social status, which was measured using the MacArthur Subjective Social Status Scale [29].

The Subjective Social Status instrument was designed to capture an individual’s perceived position in the social hierarchy [29,30]. Using a symbolic ladder with rungs ranging from 1 to 10, participants are asked to place themselves in relation to how they compare with others. The top of the ladder (number 10) represents people with the most money and prestige, whereas the bottom equates to what it means to be at the bottom of society [29,30]. In addition of using objective indicators to examine participants’ socioeconomic status (e.g., education, income), a growing number of studies have included the MacArthur Subjective Social Status Scale, as this measurement has demonstrated to relate with physical and mental health outcomes among racial/ethnically diverse populations, including samples of low-income adults of Mexican origin [30,31,32].

### 2.3. Statistical Analysis 

The associations of family lifestyle behaviors with children’s working memory were examined using multivariable linear regression tests. Associations were estimated with the overall FNPA score and with the score for each behavioral construct. Model 1 adjusted for children’s age and sex and primary caregiver’s age, education, and subjective social status. Model 2 adjusted for the covariates included in Model 1 and children’s BMI-P. In addition, consecutive adjusted multivariable linear regression tests were carried out to test the associations of each item in the FNPA with the child’s working memory to assess if the association was driven by a specific family behavior. Participants with missing data were excluded from analyses (*n* = 2). All tests were conducted with STATA v.16.1 (StataCorp, College Station, TX, USA).

## 3. Results

Table 2 introduces the descriptive characteristics of the children and caregivers included in the study sample (*n* = 85). Children’s ages ranged from 4 to 6 years (*M* = 4.58, 0.58). Of the children, 52% were female, 6% were underweight, and 33% had overweight or obesity. Primary caregivers were mostly children’s mothers (*n* = 78), but several fathers (*n* = 5) and grandmothers (*n* = 2) also participated. On average, children recalled two of the four words (*M* = 2.24, 0.99) following the timed distraction.

Table 3 introduces the results of the association of children’s working memory with their family lifestyle behaviors. A test of the two items included in the construct of family meal practice (i.e., frequency of breakfast intake and frequency of family mealtimes) revealed that frequency of breakfast intake was significantly associated with child’s working memory (β = 0.56, *p* = 0.013), but frequency of family mealtimes was not (β = 0.12, *p* = 0.465). The association with breakfast intake remained significant after controlling for children’s weight status (β = 0.57, *p* = 0.013). Children’s BMI-P was not significantly associated with children’s working memory in any of the tests performed. No other family lifestyle behavior was significantly associated with children’s working memory.

## 4. Discussion

This exploratory study indicated that frequent breakfast intake was associated with working memory among young Mexican children, and that this association was independent of the children’s weight status. No other family lifestyle behavior was associated with children’s working memory.

These findings confirm and extend findings from previous work that showed that skipping breakfast is associated with a decline in young children’s working memory [9,33]. For example, in a study conducted in West Africa, researchers found that feeding breakfast with a nutrition supplement increased working memory among children aged 15 months to 4 years old at risk of undernutrition [6]. Similarly, a previous experimental study on children aged 9–16 years demonstrated that skipping breakfast or drinking a glucose drink for breakfast associated with a decline in children’s ability to recall words [33]. In contrast, eating a cereal rich in complex carbohydrates for breakfast supported memory maintenance [33].

Previous studies that examined the different effects of breakfast composition on memory have shown that when compared with the intake of simple carbohydrates that yield high glycemic responses of short duration, the intake of complex carbohydrates, which result in slower glycemic responses of prolonged duration, associates with better learning performances in rats and improved verbal memory in young adults [34,35]. The contribution of complex carbohydrates to cognition was also documented in a study of young US children [36]. In a study among 6- to 8-year-old children, children who ate oatmeal (with a slower glycemic response) had better spatial memory and auditory attention than children who ate a ready-to-eat cereal, which produces a high glycemic response. In addition, 6- to 8-year-old girls also exhibited better short-term memory with oatmeal intake [36]. Similar benefits were also documented among 9- to 11-year-old children after consuming oatmeal, compared with children who consumed the ready-to-eat cereal [36]. In our study, the lack of dietary information limited our ability to further examine the association between children’s breakfast composition and their working memory. However, our findings make an important contribution by introducing evidence from the association between frequent breakfast intake and children’s working memory in a sample of 4- to 6-year-old Mexican children with differences in weight status that ranged from underweight to obesity.

It has been suggested that the benefits of breakfast intake are sensitive to children’s weight status and more evident in children at risk of underweight than in children with optimal growth [9,37]. In a previous study, researchers compared three experimental studies conducted among 9–11-year-old children [37]. Two were conducted among US children, and the third one was conducted among children at risk of underweight in Peru. Findings from this study suggested working memory is sensitive to fasting, and when compared to a sample of US children, the effect is stronger among Peruvian children at risk of underweight [37]. Differences by weight status, however, were not corroborated by a study conducted among 828 Danish 8–11-year-old children. In a sample of Danish children, researchers found that the association of breakfast intake with concentration performance was independent of children’s weight status, even when the study showed that normal weight children had higher cognitive performance than their peers with overweight or obesity [8]. In our study, the lack of an attenuating effect when adjusting for children’s BMI-P suggested that the association of breakfast intake with children’s working memory was independent of children’s weight status. Notably, unlike the study conducted with Danish children, we found no association between child BMI-P and working memory. We speculate that contextual environmental differences between children in the US and children in Peru could have influenced children’s performance in attention and working memory tests and hinder the ability to compare children across international samples. By including children with a similar environmental and socioeconomic status, our study and the study conducted in a sample of Danish children overcame these limitations [8].

Notably, we acknowledge the findings in this study of young Mexican children cannot be directly compared with the findings from Danish children. However, the underrepresentation of children from countries that are also experiencing the dual burden of the nutrition transition in studies of cognitive health and working memory limited our ability to compare the study findings with previous studies. To our knowledge, this is the first study examining the association of breakfast intake and family lifestyle behaviors with working memory in a current sample of children experiencing the dual burden of nutrition, with high prevalence and risk of both underweight and obesity [1,38,39]. The dual nutrition burden may have several implications for children’s cognitive development. Recent studies have shown iron deficiency anemia is prevalent among young Mexican children living in socioeconomic disadvantage [38]. Nutrition deficiencies, including iron deficiency anemia have the potential to negatively affect the development of children’s working memory, attention, and emerging executive functions [7,38].

We found no evidence of other family lifestyle behaviors associated with young children’s working memory. Previous studies have found that healthier dietary patterns, physical activity, limited screen time, and sufficient sleep are associated with children’s working memory, executive functions, attention, school performance, and ability to perform in developmental tests [5,8,10,40]. We speculate assessment of family, instead of individual behaviors, and the limited sample size, may explain these null findings.

Results should be interpreted with caution and in the context of several study limitations. Specifically, our findings are not generalizable and should be interpreted in the exploratory context in which they were investigated given the sample characteristics, limited statistical power, and cross-sectional design. Other limitations to consider are associated with the use of self-administered questionnaires, which introduced the potential for social desirability and recall bias. Future studies should verify these findings with a larger sample of racially/ethnically diverse children, objective measurements of family and individual health behaviors, and across different dimensions of cognition (e.g., attentional shift, language, motor skills). This study also lacked dietary information that would enable us to calculate the overall caloric intake, the nutrient content in children’s diets, or analyze the effects to working memory associated with different breakfast meals. There is a great need for affordable, culturally sensitive approaches that adequately measure the dietary intake of low-income and low literacy/numeracy diverse populations. Improvements in the translation of methods to assess dietary intake would empower researchers interested in addressing the limitations recognized in this study. Despite these limitations, this study makes an important contribution to the literature. To our knowledge, no study has examined the association of an array of family health behaviors—as opposed to individual behaviors, with young children’s working memory. In addition, this study introduces findings from a sample of low-income Mexican young children, a population underrepresented in science and health research, with differences in weight status that ranged from underweight to obesity.

## 5. Conclusions

Findings from this study suggest that feeding children breakfast regularly can introduce benefits to Mexican young children’s cognitive neurodevelopment. Further, results suggest that the benefits associated with frequent breakfast intake are not limited to children with normal or excess weight. More studies are needed to adequately characterize the relationship of family lifestyle behaviors with children’s working memory among children with varying weight status and across different childhood ages, particularly among diverse racial/ethnic families.

## Figures and Tables

**Table 1:** Constructs and items examined in the Family Nutrition and Physical Activity Screening Tool.

Family Constructs	Items Examined
Family Meals	1. How often does your child eat breakfast, either at home or at school?
	2. How often does your child eat at least one meal a day with at least one other family member?
Family Eating Practices	3. How often does your child eat while watching TV? [Includes meals or snacks]
	4. How often does your family eat “fast food?”
Food Choices	5. How often does your family use packaged “ready-to-eat” foods? [Includes purchased frozen or on-the-shelf entrees, often designed to be microwaved]
	6. How often does your child eat fruits and vegetables at meals or snacks? [Not including juice]
Beverage Choices	7. How often does your child drink soda pop or sweetened beverages? [Includes regular or diet soda pop, Kool-Aid, Sunny-D, Capri Sun, fruit or vegetable juice, caffeinated energy drinks (Monster/Red Bull), Powerade/Gatorade, etc.]
	8. How often does your child drink low-fat milk for meals or snacks? [Includes 1% or skim dairy, flavored, soy, almond, etc.]
Restrictions/Reward	9. How often does your family monitor the amount of candy, chips, and cookies your child eats?
	10. How often does your family use candy, ice cream, or other foods as a reward for good behavior?
Screen Time	11. How often does your child have less than 2 h of “screen time” in a day? [Includes TV, computer, game system, or any mobile device with visual screens]
	12. How often does your family monitor the amount of “screen time” your child has?
Healthy Environment	13. How often does your child engage in screen time in his/her bedroom?
	14. How often does your family provide opportunities for physical activity?
Family Activity	15. How often does your family encourage your child to be physically active?
	16. How often does your child do physical activities with at least one other family member?
Child activity	17. How often does your child do something physically active when he/she has free time?
	18. How often does your child participate in organized sports or physical activities with a coach or leader?
Family Schedule/Sleep Routine	19. How often does your child follow a regular routine for your child’s bedtime?
	20. How often does your child get enough sleep at night?

**Table 2:** Children and primary caregiver demographic characteristics.

Child/Caregiver Characteristics	*n* (%)/Mean, *sd*
**Child**	85
Sex	
Male	41 (48.24)
Female	44 (51.76)
Age (years)	4.58, 0.58
Child BMI-percentile for sex and age (percentiles)	60.74th, 32.02
Child weight status	
Underweight	5 (5.88)
Normal weight	52 (61.18)
Overweight	11 (12.94)
Obesity	17 (20.00)
Working Memory (words recalled)	2.24, 0.99
1 word	23 (26.44)
2 words	32 (36.78)
3 words	20 (22.99)
4 words	12 (13.79)
**Primary Caregiver**	85
Age (years)	30.92, 8.49
Education (years completed)	10.87, 10.44
Subjective social status (family)	6.32, 1.58

*sd* = standard deviation; BMI-P = body mass index-percentile.

**Table 3:** Association between family lifestyle behaviors and children’s working memory ^a^.

Family Behaviors	Model 1 ^b^				Model 2 ^c^			
	B	95% CI		*p*	B	95% CI		*p*
Overall FNPA Score	0.00	−0.03	0.04	0.799	0.00	−0.03	0.04	0.803
Child BMI-P					0.00	−0.01	0.01	0.757
Family Meals	0.24	0.00	0.49	0.054	0.24	−0.01	0.49	0.058
Child BMI-P					0.00	−0.01	0.01	0.780
Family Eating Practices	−0.06	−0.31	0.20	0.665	−0.07	−0.33	0.19	0.603
Child BMI-P					0.00	−0.01	0.01	0.647
Food Choices	−0.12	−0.45	0.21	0.464	−0.13	−0.42	0.20	0.428
Child BMI-P					0.00	−0.01	0.01	0.635
Beverage Choices	0.00	−0.18	0.19	0.998	0.00	−0.19	0.18	0.964
Child BMI-P					0.00	−0.01	0.01	0.724
Restrictions/Reward	0.03	−0.18	0.24	0.741	0.04	−0.17	0.26	0.695
Child BMI-P					0.00	−0.01	0.01	0.680
Screen Time	−0.04	−0.23	0.15	0.697	−0.04	−0.23	0.15	0.676
Child BMI-P					0.00	−0.01	0.01	0.699
Healthy Environment	−0.01	−0.08	0.06	0.793	−0.01	−0.08	0.06	0.804
Child BMI-P					0.00	−0.01	0.01	0.732
Family Activity	0.06	−0.12	0.24	0.494	0.06	−0.13	0.25	0.55
Child BMI-P					0.00	−0.01	0.01	0.893
Child activity	−0.05	−0.27	0.17	0.654	−0.06	−0.28	0.17	0.620
Child BMI-P					0.00	−0.01	0.01	0.678
Family Schedule/Sleep Routine	0.09	−0.08	0.27	0.286	0.09	−0.09	0.27	0.314
Child BMI-P					0.00	−0.01	0.01	0.886

CI = confidence interval; BMI-P = body mass index-percentile; ^a^ working memory scores were entered continuously based on the number of words recalled after the distracting task (range 0 to 4); ^b^ test adjusted for child’s sex, age, mother’s age, education, and subjective social status; ^c^ test adjusted for child’s sex, age, mother’s age, education, and subjective social status and child age and sex adjusted body mass index percentile (BMI-P).

## Data Availability

The dataset used and analyzed during this study is available from the corresponding author on reasonable request.
